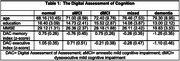# A Brief Digital Neuropsychological Protocol – IV: Relations Compared to Comprehensive Paper and Pencil Neuropsychological Assessment

**DOI:** 10.1002/alz.095672

**Published:** 2025-01-09

**Authors:** Ileana De Anda‐Duran, Lydia Bazzano, Emily Harville, Camilo Fernandez‐Alonso, Russell Banks, Sean Tobyne, Ali Jannati, John Showalter, David Bates, Alvaro Pascual‐Leone, Rodney Swenson, David J. Libon

**Affiliations:** ^1^ Tulane School of Public Health and Tropical Medicine, New Orleans, LA USA; ^2^ Tulane University School of Public Health and Tropical Medicine, New Orleans, LA USA; ^3^ Tulane University, New Orleans, LA USA; ^4^ Linus Health, Boston, MA USA; ^5^ Michigan State University, East Landing, MI USA; ^6^ Harvard Medical School, Boston, MA USA; ^7^ Boston Children’s Hospital, Harvard Medical School, Boston, MA USA; ^8^ Hinda and Arthur Marcus Institute for Aging Research and Deanna and Sidney Wolk Center for Memory Health, Hebrew SeniorLife, Boston, MA USA; ^9^ Linus Health, Waltham, MA USA; ^10^ Department of Neurology, Harvard Medical School, Boston, MA USA; ^11^ North Dakota School of Medicine, Fargo, ND USA; ^12^ University of North Dakota School of Medicine and Health Sciences, Grand Forks, ND USA; ^13^ Rowan University, Stratford, NJ USA; ^14^ Linus Health, Stratford, NJ USA

## Abstract

**Background:**

A typical paper/pencil neuropsychological evaluation to assess for mild cognitive impairment (MCI) and dementia is lengthy. There is a need for a brief, digitally administered/scored neuropsychological protocol that can differentiate patients who are cognitively normal versus MCI and dementia. This need is particularly acute with the advent of disease‐modifying medications to treat MCI and early Alzheimer’s disease (AD).

**Method:**

The Digital Assessment of Cognition (DAC) assesses memory with a 6‐word Philadelphia (repeatable) Verbal Learning Test [P(r)VLT]; executive abilities with three trials of 5 digits backward (BDST); and language/lexical access abilities with the ‘animal’ fluency test. 105 ambulatory care/memory clinic patients were assessed. DAC outcome measures include words recalled, recognition discriminability, BDST serial recall, and ‘animal fluency output; and separate episodic and executive summary indices. A portion of this sample (n = 70) underwent traditional, paper and pencil assessment.

**Result:**

Cluster analysis **(Table 1)** classified participants into groups suggesting normal (NL = 25), dysexecutive MCI (n = 25), amnestic MCI (n = 11), mixed MCI (n = 24), and dementia (n = 25) cognitive abilities. Concordance between cluster classification and clinical diagnosis using a lengthy paper/pencil protocol was 90%. The ANOVA for the episodic memory summary index found no difference between NL/dMCI groups; all other groups differed. The ANOVA for the executive index found no differences between NL/aMCI, and dMCI/mixed MCI groups; all other groups were differentiated from each other. Within‐group, dMCI patients scored lower on the executive versus the memory index (p<0.001); aMCI patients scored lower on the memory versus executive index (p<0.001); NL patients scored higher on the executive versus the memory indices (p< 0.021); and mixed MCI patients did not differ on either index. The DAC memory index was correlated with CVLT‐short form delayed free recall (r = 0.648; p< 0.001) and recognition discriminability (r = 0.625, p<0.001) scores. The DAC executive index significantly correlated with WMS‐IV Symbol Span (r = 0.620, p<0.001; Trails B (r = 0.477, p< 0.001), and letter fluency (r = 0.538, p<0.001).

**Conclusion:**

The DAC is able to identify clinically meaningful groups, showed diagnostic concordance with lengthy paper/pencil assessment, and is positively correlated with traditional paper and pencil tests. The DAC provides a reasonable means to screen for patients with putative MCI and dementia.